# Stable body sizes in soil nematodes across altitudes: The role of intrageneric variation in community assembly

**DOI:** 10.1002/ece3.70025

**Published:** 2024-07-14

**Authors:** Teng Li, Xianping Li, Lingyun Zheng, Huixin Li

**Affiliations:** ^1^ College of Resources and Environmental Sciences Nanjing Agricultural University Nanjing China

**Keywords:** altitude gradient, body size, community assembly, functional ecology, intraspecific variability, soil nematodes

## Abstract

Animal body size exhibits rapid responses to environmental variations and displays considerable variability across ecological scales, significantly influencing ecological community assembly. However, our understanding of the extent of body size variation and its responses to environmental differences within soil fauna remains limited, impeding a comprehensive grasp of soil fauna's functional ecology. Here, we aim to investigate the magnitude of intrageneric body size variation and its implications for soil nematode community assembly along an altitudinal gradient. We examined soil nematode body size responses along an altitudinal gradient spanning from 3136 to 4128 m in an alpine mountain region of the eastern Tibetan Plateau. We assessed the contributions of intra‐ and intergeneric variations in body size, both within and among communities, using individual body size values. The implications of these variations for community assembly processes were determined through phenotypic variance ratios employing permutation tests. Our analyses did not reveal statistically significant correlations between altitude and the community‐weighted mean body mass, regardless of considering intrageneric trait variation (IGTV). Approximately 15% of the variation in body size among communities and a substantial 72% of the variation in body size within communities can be attributed to IGTV. Altitude did not significantly affect IGTV within or among communities. Furthermore, our results underscored the dominant role of internal filtering within the community in governing nematode community assembly, with external filtering outside the community playing a limited role within our altitudinal range. Our findings emphasize the dominant role of body size variation within communities rather than among communities, attributable to strong internal filtering processes. These findings advance our understanding of body size variation in soil nematodes across ecological scales and highlight the pivotal role of intrageneric variation in shaping the functional ecology of soil fauna.

## Graphical abstract

Body size significantly influences species biology and ecology; yet its variations in soil fauna, like nematodes, remain unknown compared to aboveground animals. Our study addressed this gap by examining inter‐ and intrageneric variation along an altitudinal gradient and clarifying relevant community assembly processes.

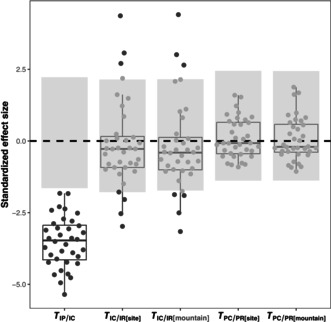

## 1 INTRODUCTION

Bergmann's rule suggests that species tend to exhibit larger body sizes at higher latitudes or altitudes, though evidence both supporting and refuting this rule exists (Blackburn et al., 1999; Meiri & Dayan, 2003; Shelomi, 2012). For ectotherms, low temperatures at high latitudes or altitudes can slow down growth rates and prolong growth periods, resulting in larger body sizes (Atkinson, 1994). However, distinct patterns in body size variation along latitude and altitude have been observed in soil fauna. For example, along altitudinal gradients, the mean body size of Onychiurinae (Collembola: Onychiuridae) decreased with increasing altitude in Changbai Mountain (Sun et al., 2020), whereas springtail (Collembola) communities at high elevations in sub‐Arctic Sweden were dominated by larger species (Bokhorst et al., 2018). Conversely, no significant variation in mean body mass was observed along elevational gradients for earthworms in the French Alps (Gabriac et al., 2023) and for nematodes across the Tibetan Plateau (Li et al., 2023). Regarding latitudinal gradients, collembolan body size was found to peak at intermediate latitudes in Europe (Ulrich & Fiera, 2010), while a global study suggested that the average community body mass of springtails was lower in polar ecosystems compared to temperate and tropical regions (Potapov et al., 2023). Despite these findings, research on body size variation in soil fauna remains limited, and the conclusions drawn from such studies are generally inconsistent along environmental gradients.

Variations in species' body size in response to environmental factors exhibit variability across ecological scales (Daufresne et al., 2009; Ohlberger, 2013). Intraspecific trait variation (ITV) within and among communities has been identified as a significant source of trait variation (Albert et al., 2010; Siefert et al., 2015; Thomas et al., 2020). Discussions regarding the general patterns of ITV in soil faunal communities have only recently begun (Bonfanti et al., 2018; Lu et al., 2023; Raposo Barros et al., 2023; Raymond‐Léonard et al., 2023). For instance, drought stress caused nematode body size decline at community, population, and individual scales (Lu et al., 2023), while earthworm body mass variation in the eastern Amazon was found to be more dependent on interspecific rather than intraspecific variation (Raposo Barros et al., 2023). However, the relative contributions of different components (e.g., species turnover, ITV) to total trait variation and their responses to natural environmental gradients remain understudied in soil fauna.

ITV not only contributes significantly to total trait variation but also exerts a notable influence on species coexistence (Jung et al., 2010; Siefert, 2012; Violle et al., 2012). Analyzing the functional structure of ecological communities has become increasingly important in deciphering the processes governing community assembly (Cornwell & Ackerly, 2009; Diaz et al., 1998; HilleRisLambers et al., 2012; McGill et al., 2006; Mouchet et al., 2010). However, many studies focused on community assembly processes based on functional traits have traditionally concentrated on species or higher taxonomic levels, often overlooking the processes acting at the individual level (i.e., without considering the effect of ITV on community assembly). Recent research has highlighted that ITV can significantly impact community assembly processes, as interactions with abiotic and biotic environments ultimately stem from individual characteristics (Jung et al., 2010; Violle et al., 2012), although the true underlying community assembly processes remain difficult to determine (HilleRisLambers et al., 2012; Kraft et al., 2015). A new framework integrating ITV into community assembly analysis introduces two distinct filters: external and internal (Violle et al., 2012). External filtering involves processes outside the community, where specific environmental conditions select specific optimal trait values at either the individual level (considering trait values of all individuals) or the species level (considering population means). Internal filtering emphasizes filtering processes within the community, often driven by density‐dependent constraints like competition, which quantifies the community‐wide overlap of ITV. Although community assembly is a prominent subject in soil faunal research (Gattoni et al., 2022; Xie et al., 2022; Zinger et al., 2019), studies considering the effects of ITV remain relatively scarce. Given the potentially significant role of ITV in shaping community assembly, its omission can lead to potentially misleading conclusions.

While ITV is crucial for understanding trait variation and its ecological implications, examining individual trait variation within coarse taxonomic resolutions is also valuable. This issue is significant because the majority of global biodiversity is composed of taxa identified at coarse levels (e.g., genus or family level), with innumerable invertebrates, including soil fauna, being representative examples (Mora et al., 2011). Intrageneric trait variation (IGTV) addresses this gap by considering trait variation within genera, providing a feasible and valuable approach for soil ecological research. Therefore, IGTV offers a new perspective on understanding the relative contributions of genus turnover and IGTV to total trait variation, as well as the strength of external and internal filtering processes along environmental gradients. This approach allows for the investigation of trait variation at a coarser taxonomic resolution, making it feasible to include a broader range of species in ecological studies.

Soil nematodes are a functionally significant group within soil fauna, known for their remarkable diversity and widespread distribution (van den Hoogen et al., 2019; Yeates, 2003). They occupy multiple trophic levels in soil food webs and exhibit considerable variation in body size (Lu et al., 2023; Mulder & Vonk, 2011; Zheng et al., 2023). For instance, omnivores and predators are typically larger than microbivorous nematodes (e.g., bacterivores and fungivores) (Bongers & Bongers, 1998; Ferris et al., 2001). Additionally, nematodes are typically identified at the genus level in soil ecology research due to a lack of expertise in species‐level identification and the predominance of unclassified species. Consequently, nematodes serve as an exemplary soil taxon for investigating variations in body size along environmental gradients and at a coarse taxonomic resolution.

Mountainous regions, characterized by varying abiotic and biotic factors, have attracted considerable attention in ecological and biogeographic research. They offer insights into how species respond to environmental differences in functional traits (Hodkinson, 2005; Jarzyna et al., 2021; Körner & Riedl, 2012; Sunday et al., 2019). Here, we conducted a field investigation of soil nematode communities along an altitudinal gradient and measured individual body sizes in each soil sample. Our primary objectives were to explore how nematode body size responds to altitude, considering intrageneric trait variation (IGTV), and to assess the role of IGTV in mediating community assembly processes. Specifically, we hypothesized that: (1) low temperatures at high altitudes exert directional selection on body size, resulting in larger body sizes in communities at higher altitudes, aligning with the empirical temperature‐size rule (Hessen et al., 2013); (2) IGTV in body size within communities should be smaller at high altitudes than at low altitudes due to stronger selective pressures reducing body size variation at high altitudes (Classen et al., 2017); and (3) external filtering may play a more substantial role than internal filtering in governing nematode community assembly along the altitudinal gradient due to pronounced changes in abiotic conditions.

## 2 MATERIALS AND METHODS

### 2.1 Study site and soil sampling

We conducted our investigation in Balang Mountain (102.86° E, 30.96° N), situated within the Wolong Nature Reserve in Sichuan Province, China. This region features a subtropical monsoon moist climate, with a mean annual temperature of 8.4°C and an annual precipitation of 861.8 mm (recorded at an altitude of 2800 m according to the Wolong Field Station).

In August 2022, we collected soil samples within a limited altitude range of 3136–4128 m at high altitudes. Six sampling sites were strategically chosen along the altitudinal gradient. These sites were situated on gentle slopes, with each site comprising six plots, approximately regularly distributed and spaced 10 m apart (Figure S1). The selection aimed to maximize altitudinal intervals while minimizing logistical constraints at higher altitudes, ensuring relatively homogeneous and flat areas for soil sampling. Vegetation transitioned from woodland (above 3000 m) to bushland and meadow (above 3500 m), and finally, to alpine meadow (above 4000 m) (Li et al., 2024). The sampling procedure involved initially removing litter, roots, and stones. Subsequently, five soil cores were obtained from each sampling plot, positioned at the four corners and the center within a 1 × 1 m square (Figure S1). Each core measured 5 cm in diameter and extended to a depth of 10 cm. To ensure homogeneity, soil cores from each plot were thoroughly mixed. All collected soil samples were promptly stored on ice in the field and transported to the laboratory. In total, 36 soil samples were gathered across the altitudinal gradient.

### 2.2 Soil nematode extraction and identification

Soil nematodes were extracted using a modified Bergmann method, based on 50 g of fresh soil (Liu et al., 2008). Approximately 100 individuals were randomly selected from each sample and identified at the genus level based on morphological characteristics (e.g., body shape, size, and head shape) using an Olympus BX50 microscope at magnifications ranging from 400× to 1000×. Nematodes were classified into one of four trophic groups (bacterivore, fungivore, herbivore, and omnivore‐predator) according to Yeates et al. (1993) and the Nemaplex database (http://nemaplex.ucdavis.edu/). To calculate the body mass of each nematode individual (fresh weight, μg), we measured the body length (*L*) and the greatest width of diameter (*D*) of each identified individual using the Motic Images Plus 3.0 software from microscope images. The body mass was then calculated using the formula *W* = (*L* × *D* ^2^)/(1.6 × 10^6^) (Ferris, 2010). The body mass was log‐transformed to improve the normality for further analyses (Figure S2).

### 2.3 Statistical analyses

Community‐weighted mean (CWM) body mass was determined by summing the body mass of taxa within the community, weighted by their relative abundance (Andriuzzi et al., 2020; Liu et al., 2015). To account for IGTV in body mass, two types of measurements were utilized for each genus. First, specific mean body mass was determined by averaging the body mass values of all individuals of a given genus within a specific plot, accounting for IGTV among communities. Second, fixed mean body mass was calculated by averaging the body mass values of all individuals of a given genus across all plots, without considering IGTV (Jung et al., 2010). Linear mixed regression models were employed to examine the relationships between CWM based on specific mean body mass and CWM based on fixed mean body mass with altitude. Site identity was treated as a random effect to address potential nonindependence among plots. The standardized regression coefficients of the mixed models were used to assess the strength of the correlation between CWM and altitude. Additionally, we evaluated correlations between the mean body sizes of widely distributed genera (e.g., occurring at all altitudes) and altitude using mixed models. To account for the increase in Type I error rate during multiple comparisons, the significance of altitude on body size was adjusted using the false discovery rate (FDR) method.

To assess the relative contribution of IGTV to total trait variation both among and within communities, we adopted the following approaches. For IGTV among communities, we decomposed the variation in specific body mass into three sources: changes in community composition (turnover), intrageneric variability, and their covariation. Intrageneric variability in body mass is defined as the difference between specific and fixed body mass, while the difference in fixed body mass represents the contribution of turnover. The relative importance of these sources and their responses to altitude were analyzed using parallel ANOVAs following the approach of Lepš et al. (2011). Regarding IGTV within communities, we initially calculated the abundance‐weighted intergeneric and intrageneric trait variances for each community. The relative contribution of IGTV within communities was quantified as the ratio of the IGTV over the total within‐community trait variance (sum of intergeneric and intrageneric trait variance) according to the methodology of Siefert et al. (2015).

To further investigate the variation in body size across multiple nested scales, we decomposed the variance in body size into five nested levels: site, plot, trophic group, genus, and individual. We fitted a linear mixed model with all these nested levels treated as random factors, using the restricted maximum‐likelihood method. Subsequently, a variance component analysis was conducted on this model to determine the variances in nematode body mass across these nested scales (Messier et al., 2010). This approach differs from previous calculations of IGTV among and within communities by encompassing both plot and site scales.

To investigate the community assembly processes influenced by IGTV, we employed T‐statistics (phenotypic variance ratios) that originally consider intraspecific variation relative to interspecific variation (Violle et al., 2012). As nematodes were identified at the genus level, we slightly modified the calculations of the T‐statistics to match our data structure while retaining the original concept and methodology. *T*_IP/IC_ represents the ratio of variation in body mass among individuals within a genus to variation in body mass among individuals within a community. *T*_IC/IR_ reflects the ratio of variation in body mass among individuals within a community to the variation in body mass among individuals within a regional pool, while *T*_PC/PR_ indicates the ratio of variation in genus mean body mass within a community to the variation in genus mean body mass within a regional pool (Violle et al., 2012). These variance ratios can be used to quantify the impact of internal and external filtering on a given community at different organizational scales. *T*_IP/IC_ quantifies the strength of internal filtering, referring to filtering processes within the community. On the other hand, *T*_IC/IR_ and *T*_PC/PR_ quantify the strength of external filtering, which pertains to filtering processes outside the community. *T*_IC/IR_ focuses on external filtering processes acting on individuals, while *T*_PC/PR_ represents external filtering processes acting on genus averages (Violle et al., 2012). To assess the significance of these T‐statistics, we generated null models through random resampling of individual trait values within the community (*T*_IP/IC_), resampling individual trait values from the regional pool without replacement (*T*_IC/IR_), or resampling genus‐level values from the regional pool without replacement (*T*_PC/PR_). To address potential sensitivity to the choice of regional pool, we conducted calculations and null models by assigning the regional pool of each plot to either the entire mountain or the corresponding site. This resulted in the generation of five T‐statistics: *T*_IP/IC_, *T*_IC/IR[mountain]_, *T*_PC/PR[mountain]_, *T*_IC/IR[site]_, and *T*_PC/PR[site]_, where the subscripts “mountain” and “site” indicated the regional pools at the levels of the entire mountain and individual sites, respectively. The standardized effect size (SES) of each T‐statistic was calculated based on 999 permutations (Taudiere & Violle, 2016). Additionally, we compared *T*_IC/IR_ with *T*_PC/PR_ to determine whether filtering processes predominantly act on genus averages (*T*_IC/IR_ < *T*_PC/PR_) or on individual values (*T*_IC/IR_ > *T*_PC/PR_) at different regional pools (Violle et al., 2012). To account for potential nonindependence among plots, we employed mixed models based on the combined data of *T*_IC/IR_ and *T*_PC/PR_, with plot and site treated as random factors, to test the significance of the difference between the paired *T*_IC/IR_ and *T*_PC/PR_ values at two regional pools.

All the statistical analyses were conducted in R version 4.0.3 (R Core Team, 2020).

## 3 RESULTS

### 3.1 CWM body size along altitude

**FIGURE 1:**
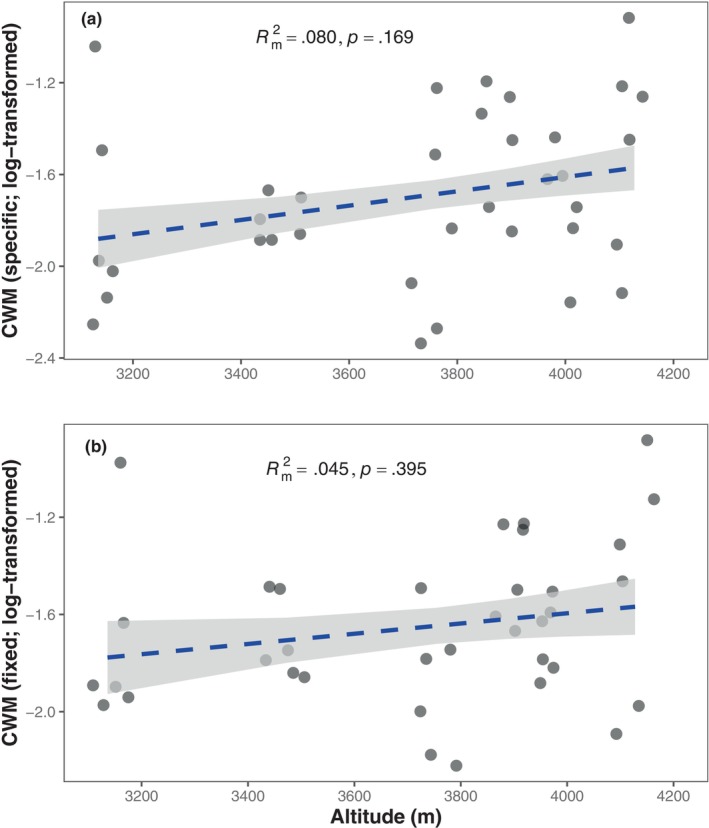
Community‐weighted mean (CWM) body mass of soil nematodes along altitude. (a) Specific mean body mass, calculated by averaging the body mass values of all individuals of a given genus within each plot, considering intrageneric variability. (b) Fixed mean body mass, calculated by averaging the body mass values of all individuals of a given genus across all plots, without considering intrageneric variability. Dashed lines indicate nonsignificant relationships between CWM body mass and altitude based on mixed models with site identity as a random factor, while gray ribbons represent the confidence intervals of the regression lines. $R_{m}^{2}$ represents the variance explained by the fixed effect (i.e., altitude) using the *r.squaredGLMM* function in the R package MuMIn. The points were jittered to avoid overlap using the *geom_jitter* function in the ggplot2 package.

**FIGURE 2:**
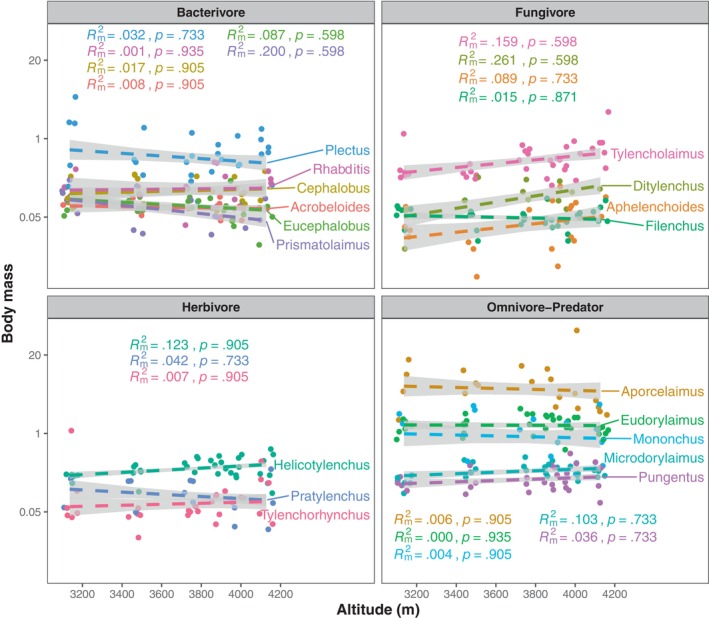
The relationships between nematode body mass and altitude for representative genera (i.e., the genera occurring at all altitudes). Dashed lines represent nonsignificant relationships between the body mass of each genus with altitude based on mixed models with site identity as a random factor. $R_{m}^{2}$ represents the variance explained by the fixed effect (i.e., altitude) for each genus with a *p*‐value adjusted using FDR. The points were jittered to avoid overlap using the *geom_jitter* function in the ggplot2 package.

In total, 37 genera were collected in this study, with the number of genera ranging from 11 to 22 and a mean value of 16.17 (Table S1). Overall, we did not find significant associations between CWM body size and altitude, regardless of whether the IGTV was considered (specific mean body mass; standardized regression coefficient *β* = .288, *p* = .169) or not (fixed mean body mass; *β* = .220, *p* = .395) (Figure 1). Additionally, different genera exhibited varying responses to altitude (Figure 2). However, among those widely distributed genera, we observed consistent patterns of no correlation between mean body size and altitude for all genera after adjusting the *p*‐values using the FDR method (Figure 2).

### 3.2 Intra‐ and intergeneric variation in body size

By decomposing the total body size variation across communities into turnover, intrageneric variability, and their covariation, we identified that a significant proportion of body size variation (78.18%) was attributed to turnover, representing changes in community composition. In contrast, a relatively smaller fraction (15.22%) was linked to intrageneric variability (Table 1). Additionally, we observed a small positive covariation (6.60%) between turnover and intrageneric variability. In line with the trends observed in CWM along altitude, we found that less body size variation was associated with altitude (8.30%). Altitude appeared to have a slightly greater effect on turnover (3.79%) compared to intrageneric variability (0.87%), although the magnitudes were generally small (Table 1).

**TABLE 1:** Decomposition of body size variation among communities into turnover, intrageneric variability, and their covariation along altitude.

	Turnover		Intrageneric variability		Covariation		Total	
	Variability	Proportion	Variability	Proportion	Variability	Proportion	Variability	Proportion
Altitude	0.1781	0.0379	0.0411	0.0087	0.1711	0.0364	0.3904	0.0830
Error	3.5001	0.7439	0.6751	0.1435	0.1393	0.0296	4.3145	0.9170
Total	3.6782	0.7818	0.7162	0.1522	0.3105	0.0660	4.7049	1.0000

*Note*: Variability corresponds to the total sum of squares of each component, and proportion means the relative contribution of each component to total variation.

**FIGURE 3:**
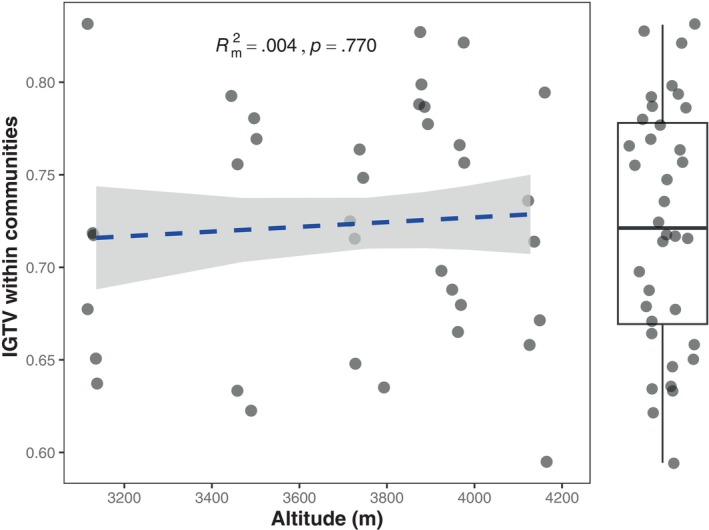
The relationship between intrageneric body mass variation (IGTV) within communities and altitude. The margin plot displays boxplots illustrating intrageneric trait variation within communities. The dashed line indicates no significant relationship between IGTV and altitude based on a mixed model with site identity as a random factor. The gray ribbon represents the confidence interval of the regression line. The points were jittered to avoid overlap using the *geom_jitter* function in the ggplot2 package.

Regarding IGTV within communities, our study revealed remarkably high values ranging from 59.52% to 83.14%, with a mean value of 72.35% across all communities (Figure 3). However, we did not detect a significant correlation between IGTV within communities and altitude (*β* = .067, *p* = .770) (Figure 3). Our analysis of variance in body size among all individuals across nested scales showed that the majority of the variation stemmed from the genus level (59.80%), followed by individual (22.85%), trophic group (17.32%), and minimal contributions from the site (0.04%), and plot (0.00%) levels (Figure S3).

### 3.3 Body size IGTV‐mediated community assembly

**FIGURE 4:**
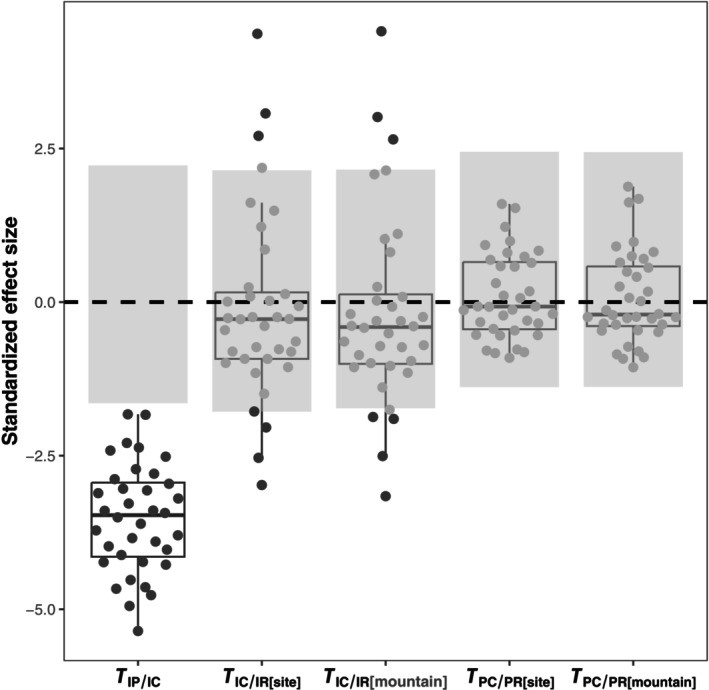
Standardized effect size (SES) of five T‐statistics for nematode body mass. *T*_IP/IC_ represents the ratio of within‐genus variance to total within‐community variance; *T*_IC/IR[site]_ and *T*_IC/IR[mountain]_ represent community‐wide variance relative to the total variance in the regional pool of each responding site and the entire mountain; *T*_PC/PR[site]_ and *T*_PC/PR[mountain]_ represent intercommunity variance relative to the total variance in the regional pool of each responding site and the entire mountain. Each point represents the SES value for one community, with gray points indicating no significant difference from the null distribution for each community. The gray rectangle delineates the mean values of the confidence interval of SES values obtained from null models across all plots for a given T‐statistic.

The mean values for the observed *T*_IP/IC_, *T*_IC/IR[site]_, *T*_IC/IR[mountain]_, *T*_PC/PR[site]_, and *T*_PC/PR[mountain]_ were 0.252, 0.981, 0.960, 1.02, and 1.01, respectively. Results from null models revealed that the observed *T*_IP/IC_ values across all communities significantly deviated from null values (mean SES value = −3.518, ranging from −5.354 to −1.826). However, the observed *T*_IC/IR[site]_ values (mean SES value = −0.107, ranging from −2.978 to 4.367), *T*_IC/IR[mountain]_ values (mean SES value = −0.185, ranging from −3.159 to 4.408), *T*_PC/PR[site]_ values (mean SES value = 0.092, ranging from −0.908 to 1.597), and *T*_PC/PR[mountain]_ values (mean SES value = 0.067, ranging from −1.062 to 1.880) were not significantly different from their respective null values (Figures 4 and Figure S4). Furthermore, neither the observed T‐statistics nor their standardized effect sizes exhibited correlations with altitude (all *p* > .05; Figures S4 and S5). A comparison of *T*_IC/IR_ and *T*_PC/PR_ values indicated that the filtering strength was not significantly different for genus averages and individual values (mixed model regression coefficient, *β* = .049, *p* = .245 with a regional pool encompassing the entire mountain; *β* = .042, *p* = .311 with a regional pool specific to the site).

## 4 DISCUSSION

Variation in body size is a common phenomenon along environmental gradients and in response to global changes. Intraspecific trait variation (IGTV), often overlooked in soil faunal studies, can be a significant source of body size variation within and among communities, influencing community assembly processes. Here, we quantified the extent of IGTV along an altitudinal gradient and assessed its impact on community assembly, using soil nematodes as a model organism. Our findings revealed a consistent community‐weighted mean body size of soil nematode communities across varying altitudes. While IGTV contributed considerably to body size variation among communities, its effect was lower compared to turnover. However, within communities, IGTV exhibited a stronger influence on body size variation compared to intergeneric variation. Overall, the altitude did not significantly affect IGTV, whether at the community or within‐community levels. Moreover, our results provided evidence of strong internal filtering and weak external filtering based on intrageneric and intergeneric trait variances across hierarchical ecological levels.

Community‐weighted mean values of functional traits are frequently employed to describe species responses to environmental gradients and community‐level changes (Funk et al., 2017; Violle et al., 2014). However, many studies have traditionally utilized fixed values for each species across communities, often relying on trait data sourced from public databases (Kattge et al., 2020; McGill et al., 2006; Weiher et al., 2011; Wilman et al., 2014). This approach has faced criticism for neglecting intraspecific trait variation (Bolnick et al., 2011; Jung et al., 2010; Siefert et al., 2015). Our findings underscored the potential significance of incorporating IGTV into CWM analysis, revealing different trends depending on whether IGTV was considered. Specifically, when IGTV was accounted for, a slightly stronger effect of altitude on CWM was observed, reflecting variations in how different genera responded to changes in altitude. However, contrary to our initial hypothesis, nematode body size did not increase with rising altitude. This discrepancy may be attributed to the possibility that the environmental gradient in our study was not extensive enough to exert substantial selective pressure on nematode body size. Alternatively, soil nematodes may employ other strategies, such as dietary shifts, changes in fecundity, or adjustments in sex ratio (Klusmann et al., 2022; Majdi et al., 2019; Okada et al., 2002), rather than altering body size in response to environmental differences, as initially hypothesized. Nevertheless, the altitudinal trends in body size varied among genera, suggesting that changes in body size may be taxon‐specific. Further investigations at finer taxonomic resolutions are warranted to determine the generality of this pattern.

Through decomposing the total variation in body mass into turnover, IGTV, and their covariation across nematode communities, we revealed that turnover predominantly drives body size variation among communities. Although few studies have focused on IGTV as we did, we anticipated potential context‐dependent variability in the relative contributions of different components to total trait variation. This expectation is based on studies focused on plant traits along elevation gradients, which have suggested different patterns among traits and regions (Kuppler et al., 2020; Luo et al., 2016; Roos et al., 2019; Westerband et al., 2021). The substantial influence of turnover on total trait variation in the current nematode study can be attributed to significant changes in nematode community composition along the altitudinal gradient (Figure S6). However, this approach may have limited power due to its simplicity in variance decomposition using parallel simple ANOVAs. Further improvements in the decomposition of trait variability across multiple error levels will enhance our understanding of the relative contributions of species turnover and intraspecific/intrageneric variability in more diverse experimental and sampling designs. Notably, IGTV within communities accounted for nearly three fourths of the total variation, a proportion significantly higher than the mean value of 25% of intraspecific trait variation reported in a meta‐analysis involving plant communities and multiple traits (Siefert et al., 2015). This deviation is likely due to our use of a coarser taxonomic resolution, specifically, genus rather than species. It is expected that there should be more extensive trait variation among different species compared to within a single species. Additionally, our hypothesis of a decrease in IGTV, either within or among communities, along the altitudinal gradient was not supported by our findings. This indicates that altitude and associated abiotic factors may not exert strong filtering effects on intrageneric body size variation among nematodes in our ecosystem. Furthermore, the results obtained from the mixed model analysis across nested levels further underscored that body size variation at the genus and individual levels, rather than at the site and plot levels, made the most significant contributions to the total variation among all individuals. This highlights the limited impact of environmental factors on nematode body size variation. Given the substantial role of IGTV in total trait variation, studies using mean body size or biomass based on measurements or databases at the genus level may not fully characterize the ecological processes acting on nematode communities (Li et al., 2020, 2023).

In contrast to previous studies that focused on soil nematode community assembly based on phylogenetic relationships among taxa (Kang et al., 2022; Zou et al., 2023) or by partitioning variation in community composition into different variable sets (Gattoni et al., 2022; Kang et al., 2022), our study introduces a novel perspective by incorporating IGTV into community assembly analysis. Our investigation revealed that the community‐wide overlap of IGTV was significantly smaller than expected under null models, indicating substantial internal filtering processes acting on individuals within the community. This suggests that strong biotic interactions within communities may play a pivotal role in reducing local intrageneric variation (Violle et al., 2012). However, we did not detect significant external filtering at either the genus averages or individual values across different regional pools. This may be due to the limited altitudinal range, the potential insensitivity of nematode body size to environmental gradients, or the proximity between plots within a site in our study. Although these findings diverged from our third hypothesis that external filtering would outweigh internal filtering in governing soil nematode communities, they were consistent with some previous studies that also identified robust internal filtering using T‐statistics (Khalil et al., 2019; Taudiere & Violle, 2016; Zorger et al., 2019). Furthermore, we did not observe significant trends in these filters, including both internal and external filtering, along the altitudinal gradient. This suggests that altitude may not directly govern soil nematode community assembly through filtering based on body size. However, given that environmental selections and biotic competitions can yield similar community structures, caution should be exercised in further interpreting external and internal filtering mechanisms (HilleRisLambers et al., 2012; Kraft et al., 2015).

In contrast to studies focused on ITV within well‐classified taxonomic groups such as plants and large animals, our focus on IGTV might seem coarse and less informative. However, several factors underscore the value of our study. First, as previously mentioned, coarse classification is common for many taxonomic groups. Our study not only provides specific findings but also aims to inspire experts working on other coarsely classified taxa, thus offering a broader perspective on species trait variation. Additionally, while the contributions of IGTV to total trait variation and the effects of external selections at the genus level have not been explicitly tested, our results indicate a substantial role of IGTV and a dominant effect of internal filtering within the genus. Furthermore, our approach and findings may lead to new hypotheses. For example, whether genera with larger IGTV have broader range sizes (Rixen et al., 2022), whether the magnitude of IGTV exhibits phylogenetic conservatism (Ives et al., 2007), and whether high IGTV enhances community resistance to environmental changes (Jónsdóttir et al., 2023) are all questions worth exploring. Answers to these questions will expand our understanding of the ecology, evolution, and biogeography of these less‐studied taxa. Nonetheless, the effects of differences among species and the uneven distribution of species within different genera on ecological and evolutionary processes still need investigation. We believe further advancements in species classification will enhance our comprehension of trait variation across multiple biological scales and unveil overlooked processes within species.

It is essential to acknowledge several limitations that could affect the robustness of our findings. Firstly, although body size is a crucial functional trait influencing species fitness and environmental responses, focusing solely on this trait may not comprehensively elucidate all ecological processes related to functional traits. Further research incorporating additional traits and examining multiple traits across diverse environmental gradients is critically needed to validate our findings thoroughly. Additionally, this study is specific to a high altitudinal range in a specific region and focuses on a particular taxon. Future studies across different ranges, regions, taxa, and spatial scales are critical. While these constraints may compromise the generalizability of our conclusions, this study represents an initial exploration of the ecological roles of IGTV in soil nematode ecology.

In conclusion, our integration of IGTV into the study of nematode community ecology has provided valuable insights into soil nematode body size responses to environmental factors and their impact on community assembly. Our study highlighted the significant role of IGTV within communities and its substantial contribution to variations in nematode body size among communities. Furthermore, our research revealed that internal filtering, rather than external filtering, predominantly governs the assembly of nematode communities. However, the influence of altitude on IGTV and the assembly processes of soil nematode communities appears to be limited. In summary, our case study not only elucidates the patterns of body size variation across ecological scales within soil nematodes but also highlights the unignorable influences of intrageneric trait variation on nematode responses to environmental conditions and their role in community assembly.

## AUTHOR CONTRIBUTIONS

**Teng Li:** Formal analysis (equal); writing – original draft (equal); writing – review and editing (equal). **Xianping Li:** Conceptualization (equal); formal analysis (equal); funding acquisition (equal); investigation (equal); writing – original draft (equal); writing – review and editing (equal). **Lingyun Zheng:** Investigation (equal). **Huixin Li:** Conceptualization (equal); funding acquisition (equal); supervision (equal); writing – review and editing (equal).

## CONFLICT OF INTEREST STATEMENT

The authors declare no conflict of interest.

## Supporting information


Appendix S1

## Data Availability

Data for this article are archived in the Figshare Digital Repository at https://figshare.com/s/341477db52bea117a367.
